# Impacts of Climate Change on Vector Borne Diseases in the Mediterranean Basin — Implications for Preparedness and Adaptation Policy

**DOI:** 10.3390/ijerph120606745

**Published:** 2015-06-15

**Authors:** Maya Negev, Shlomit Paz, Alexandra Clermont, Noemie Groag Pri-Or, Uri Shalom, Tamar Yeger, Manfred S. Green

**Affiliations:** 1 School of Public Health, University of Haifa, Haifa 3498838, Israel; E-Mails: maya.negev@gmail.com (M.N.); nomiroie@gmail.com (N.G.P.O.); manfred.s.green@gmail.com (M.S.G.); 2 Department of Geography and Environmental Studies, University of Haifa, Haifa 3498838, Israel; 3 The Arava Institute for Environmental Studies, Kibbutz Ketura 8884000, Israel; E-Mail: alexclermont@gmail.com; 4 Division of Pest Surveillance & Control, Israel Ministry of Environmental Protection, P.O. Box 34033, Jerusalem 95464, Israel; E-Mails: Urys@sviva.gov.il (U.S.); TamarY@sviva.gov.il (T.Y.)

**Keywords:** climate change, vector-borne diseases, Mediterranean, adaptation policy

## Abstract

The Mediterranean region is vulnerable to climatic changes. A warming trend exists in the basin with changes in rainfall patterns. It is expected that vector-borne diseases (VBD) in the region will be influenced by climate change since weather conditions influence their emergence. For some diseases (*i.e.*, West Nile virus) the linkage between emergence andclimate change was recently proved; for others (such as dengue) the risk for local transmission is real. Consequently, adaptation and preparation for changing patterns of VBD distribution is crucial in the Mediterranean basin. We analyzed six representative Mediterranean countries and found that they have started to prepare for this threat, but the preparation levels among them differ, and policy mechanisms are limited and basic. Furthermore, cross-border cooperation is not stable and depends on international frameworks. The Mediterranean countries should improve their adaptation plans, and develop more cross-sectoral, multidisciplinary and participatory approaches. In addition, based on experience from existing local networks in advancing national legislation and trans-border cooperation, we outline recommendations for a regional cooperation framework. We suggest that a stable and neutral framework is required, and that it should address the characteristics and needs of African, Asian and European countries around the Mediterranean in order to ensure participation. Such a regional framework is essential to reduce the risk of VBD transmission, since the vectors of infectious diseases know no political borders.

## 1. Introduction

According to the recent IPCC report [1], the climate system warming is clear and unequivocal. Although climate change is a complex phenomenon, it is well established that it influences the emergence of vector-borne diseases (VBDs) such as malaria, West Nile fever or dengue [2,3,4,5,6]. VBDs are dynamic systems with complex ecology, which tend to adjust continually to environmental changes in multifaceted ways. Although diverse factors impact the distribution of VBDs, climate is a major driver that influences their epidemiology. The life-cycle dynamics of the vector species, pathogenic organisms and the reservoir organisms are all sensitive to weather conditions, which affect the survival and reproduction rates of the vectors, their habitat suitability, geographical distribution and abundance, and impact their seasonal intensity and temporal activity. Additionally, climatic factors affect the rates of development, reproduction and survival of pathogens within the vectors [6,7,8]. Following the WHO evaluations, since climatic conditions strongly affect diseases transmitted through insects, climate change is likely to alter the geographic range of VBDs, to lengthen their transmission seasons [9] and to affect beyond their current seasonal patterns. 

In recent years, several outbreaks of different VBDs have been documented in the Mediterranean basin region. For some of them, linkages with local climatic changes have been already proved [10,11]. For others, it is reasonable to assume that the recent observed climatic trends in the basin will contribute to their transmission potential in the region [8,12]. 

The Mediterranean region (the Mediterranean Sea and its surrounding areas) lies in a transition zone between the arid climate of North Africa and the temperate and rainy climate of central Europe, as well as between the Atlantic Ocean and the land mass of Asia. The climate of the basin is determined by the interaction between mid-latitude and sub-tropical circulation regimes and a complex morphology of mountain chains and land–sea contrasts [13]. This makes the Mediterranean a potentially vulnerable region to climatic changes [14]. Moreover, the region has been identified as one of the main climate change hotspots and one of the most responsive areas to global warming [13,15,16]. Since the 1960s, the Mediterranean region has become warmer, with a significant increase in the frequency, intensity and duration of heat waves. In addition, the basin is characterized by a reduction in the availability of potable water as a result of decrease in the total amount of precipitation, changes in rainfall patterns and water overuse by the growing population. In the Mediterranean basin, mutual enhancement (positive feedback) between droughts and heat waves has been identified [1,17,18,19]. 

The area is populated by over 500 million people and includes 27 countries in North Africa, western Asia, and southern Europe [13]. With significant gaps in the socio-economic levels among the Mediterranean countries, particularly between the North (Europe) and South (Africa), together with population density and increased water demand, the vulnerability of the region under changing climatic conditions is increasing.

In this paper we have two main aims: first, to briefly highlight the knowledge regarding the transmission potential of the predominant VBDs (transmitted by insects) in the Mediterranean basin, that have a real potential to be impacted by climate change. For part of them, linkages with the recent climatic fluctuations in Mediterranean countries have already been proved [11,20], for others, projections (e.g., of the WHO) indicate that among other factors, climatic suitability for the vectors will increase in new regions as a result of climate change [21,22,23,24]. The second aim is to analyze and compare adaptation policies in a representative selection of Mediterranean countries, according to relevant policy categories: monitoring and surveillance, environmental management, health system preparedness and public education. In addition, we identify existing mechanisms for regional collaboration regarding environmental and health issues. In the last section of the paper, we make recommendations for VBD adaptation in the Mediterranean, at the national and regional levels. 

## 2. Vector (Insect)-Borne Diseases and Climatic Factors

The ecology, development, behaviour, and survival of insects and the transmission dynamics of the diseases they transmit are strongly influenced by climatic factors. Temperature, rainfall, and humidity are especially important, but others such as wind can also be significant. The same factors also play a crucial role in the survival and transmission rate of the pathogens. The main parameter that affects the rate of multiplication in the insect is temperature [25]. When the temperature increases, it tends to cause an upsurge in the growth rates of mosquito populations, decrease the interval between blood meals, shorten the incubation time from infection to infectiousness in mosquitoes and accelerate the virus evolution rate [26,27].

It is known that above-average precipitation can lead to a higher abundance of mosquitoes and increase the potential for disease outbreaks in humans [28]. This was shown for several VBD outbreaks, including West Nile virus [29], dengue [30] and malaria [31]. However, although the patterns of disease incidence can be influenced by the rainfall amount, the response might change over large geographic regions, depending on differences in the ecology of mosquito vectors [6,28]. For instance, heavy rainfall increases the standing water surface which is necessary for mosquito larval development [32]. On the other hand, drought conditions can facilitate population outbreaks of some species of mosquitoes because the drying of wetlands disrupts the aquatic food-web interactions that limit larval mosquito populations [33,34,35]. 

Since the Mediterranean is undergoing a warming trend with an increase in warm days and nights, longer and warmer summers, an increase in the frequency and the severity of heat waves and a reduction in rainfall amounts, it is expected that VBDs will be exacerbated by climate change [36]. Moreover, most cities in the Mediterranean are compact and densely populated. Air conditioning is used in regions with advanced socio-economic level, but as part of the local mentality windows remain open for most of the hot months. Many activities, particularly social gatherings, occur in outdoor locations such as shaded balconies, courtyards, and outdoor restaurants—all ideal for contact with the vector. While warmer summers extend the potential range of the disease, the poorer countries, particularly in North Africa and the Levant, would be at highest risk [25]. Currently, the main vector-borne diseases, transmitted by mosquitoes and potentially influenced by the changing climate in the Mediterranean basin, are:

### 2.1. West Nile Fever 

The West Nile virus is a vector-borne pathogen of global importance since it is the most widely distributed virus of the encephalitic *Flavivirus* spp. Mosquito species from the genus *Culex* (family *Culicidae*) are the primary amplification vectors and also act as bridge vectors. The enzootic cycle is driven by continuous virus transmission to susceptible bird species through adult mosquito blood meal feeding, which results in virus amplification [37]. 

During the last years, West Nile Fever (WNF) cases in humans have increased in several Mediterranean countries. For example, in Israel, a severe upsurge occurred during the hot summer of 2000 [38] and again in the extremely warm summer of 2010 [39]. A change in the seasonality of the disease was observed, as the outbreaks began earlier in the year [40]. An outbreak of WNV infection first occurred in Central Macedonia in northern Greece in the summer of 2010 [41]. During the same period, cases in humans were also reported in other Mediterranean countries: Turkey, Italy and Spain (together with other locations, mainly in Eurasia). Additionally, WNV was detected in horses in Greece, Italy, Gibraltar and Morocco [11,37]. A study by Paz *et al.* [11] found that uncharacteristically elevated temperatures during the summer of 2010 correlated with the WNF upsurge in humans. According to the World Meteorological Organization [42], such events become much more common—the summer of 2010 was not only one of the top three warmest summers ever recorded, but part of a continuing warming trend of Europe.

Since 2010, all subsequent years (2011–2014) have been characterized by the re-emergence of WNV within the same countries [43]. Recent research by Tran *et al.* [20] analyzed the status of infection by WNV in Europe and its neighbouring countries in relation to environmental and climatic risk parameters. The anomalous temperatures in July were identified as one of the main risk factors.

### 2.2. Dengue

Dengue is a mosquito-borne disease in humans, caused by a virus of the *Flavivirus* genus, *Flaviviridae* family. Dengue fever is transmitted through bites of *Aedes* mosquitoes and is one of the most prevalent vector-borne diseases in the world, affecting about 390 million people each year [44]. Transmission of the dengue virus is sensitive to climate. Temperature, rainfall and humidity affect the breeding cycle, survival and biting rate of the mosquito vectors, while temperature in particular favors the rapid development of the vector (which is highly sensitive to climate conditions), increases the frequency of blood meals, and reduces the extrinsic incubation period [9,45]. It was noted by the WHO that the recent geographical distribution of dengue depends strongly on climatic and socioeconomic variables. The WHO models predict that climate change would contribute to an expansion of the current dengue distribution (but socioeconomic developments may counteract this change) [24]. 

*Aedes* spp. mosquitoes are widely distributed in Africa and can serve as dengue virus vectors. In Europe, *Aedes albopictus* arrived in Albania in 1979, and made its way to Italy in the early 1990s via trade in used tires. Today, it is present mainly in the northwest Mediterranean basin [46,47]. According to the ECDC evaluations, future expansion of the vector could be further facilitated by climate change, as altered warming and precipitation patterns might increase the number of suitable niches [8,48].

During the years 2008–2012, dengue fever cases were reported in several Mediterranean (and Adriatic) countries: Greece, Croatia, Italy, Malta, France, Spain and Portugal [43,49,50]. Although most cases were probably imported, in 2010 local transmission of dengue was reported in both Croatia and France [51]. Today, there is an apparent threat of dengue outbreaks in the Mediterranean European countries [12,47]. 

### 2.3. Chikungunya

Chikungunya virus is transmitted to people through *Aedes aegypti* and *Aedes albopictus* mosquito bites (the same mosquitoes that transmit dengue virus) [52]. In 2007, first transmission in Mediterranean Europe was reported from northeastern Italy. During the period between 2008 and 2012 imported cases were reported in several countries in the basin including Greece, Italy, France and Spain [43]. Gould and Higgs [21] noted that if global climate change continues, *A. albopictus* and *A. aegypti* will disperse beyond their current geographic boundaries, since temperature plays a very significant role in the development (and mortality rates) of *Aedes albopictus* [53]. As *A. albopictus* is currently present in the region, Chikungunya outbreaks may be caused in the northwestern Mediterranean [54] under favorable climatic conditions [55].

### 2.4. Malaria

Malaria is caused by infection with a protozoan of the genus *Plasmodium*, transmitted through the bite of an infected *Anopheles* mosquito. Although the disease is determined by socioeconomic, environmental and behavioural factors, the potential for malaria transmission is intricately connected to meteorological conditions [8]. During the years 2008–2012, malaria cases were reported in several Mediterranean countries: Cyprus, Greece, Malta, Spain and Portugal. While most malaria cases were reported as imported, in 2012 twenty-two cases from Greece and one from France were reported as not imported [43]. In 2012 in the eastern Mediterranean and North Africa, malaria cases were reported in Lebanon, Egypt, Libya, Tunisia and Morocco. According to the WHO, all cases were imported with no local transmission [56]. Since dominant or potentially important malaria vectors exist in the area [57], global climate change creates the potential, albeit relatively small, for the reappearance of malaria in countries where it was previously eradicated [8].

### 2.5. Leishmaniasis

Leishmaniasis is a vector-borne disease, caused by infection of *Leishmania* parasites, which are transmitted by the bite of infected female *Phlebotomine* spp. sandflies. In the Mediterranean basin, *Leishmania major* and *Leishmania tropica* cause cutaneous leishmaniasis (CL), which manifests as skin sores. *Leishmania infantum* causes visceral leishmaniasis (VL) [58,59,60]. Globally, CL is more widely distributed, with about one-third of cases occurring in each of three main regions, one of them being the Mediterranean basin [61]. During the period of 2003–2008, CL cases were reported from 16 Mediterranean countries, particularly in the eastern and southern sides of the basin. On average, 85,555 CL cases per year were reported for the entire region, most of them in Algeria, Syria, Libya and Morocco. During the same period, VL cases were reported from 22 Mediterranean countries around the whole basin (on average, 875 reported cases per year for the entire region) [61]. 

Arthropod vectors such as sandflies are especially sensitive to changes in temperature and moisture. It is known that for *P. papatasi* low temperatures are often the limiting factor in determining its seasonal activity period [62]. Relatively small changes in temperature may have a large effect on their vectorial capacity. Since temperature affects the level of activity of the sandfly, it consequently affects the frequency of its blood meals [63]. At the same time, it is important to note that extremely hot and dry conditions also limit *P. papatasi* activity [64]. In a study by Boussaa *et al.* [65] in Marrakech, Morocco, the preferred temperature for *P. papatasi* ranged from 32–36 °C and flies were less active at temperatures of 11–20 °C and 37–40 °C. They also showed that although sand flies were active especially during the dry season (May to November), the activity peaks were noted during the wettest months (May and November) of the dry season [65]. 

Since shifts in temperature and relative humidity affect the survival and reproduction rates of these vectors, as a result, the intensity and temporal pattern of the vector activity may be altered.

Despite international efforts to mitigate the effects of climate change by reducing the production of greenhouse gases, it is now clear that climate change cannot be prevented. Thus, comprehensive plans are necessary to ensure effective adaptation to the different manifestations of climate change. 

Due to many uncertainties, the “no regret” approach has been proposed by international organizations. This approach is based on directing resources to preparedness measures which will have a positive impact on public health, regardless of the impact of climate change. In addition to recommending specific preparedness measures, the WHO has called for strengthening health, environmental and social systems to improve prevention, preparation and coping with climate change, and for sharing research, data, best practice and technology on climate change, environment and health [66]. 

In order to deal with these potential health threats and before the VBD develop into regional outbreaks, Mediterranean countries must develop national adaption policy, which include strategic response systems in order to be effective. Such policies should be geared towards both the prevention of the disease and the management of the events that may arise, as no vaccinations are available yet for the VBD that emerge in the Mediterranean. Since there are geographical variations in the effects of climate change and the vulnerability of populations, adaptation measures must be addressed at both local and regional levels [67].

## 3. National Adaptation Policy in the Mediterranean: The Current Situation 

In 2010, the 195 parties of the United Nations Framework Convention on Climate Change (UNFCCC) adopted the Cancun Adaptation Framework. This framework recognizes the importance of adaptation to climate change, and the parties committed to planning and implementing adaptation plans [68]. International guidelines for National Adaptation Plans include preparation for emerging threats to human health, including VBD [66,69]. 

For example, 18 out of 32 European countries have adopted a national level adaptation strategy addressing climate change impacts on human health, of which 86% discuss vector-, food-, or water-borne infections. This type of climate-induced health concern was second only to the one of heatwaves (90% of the documents), followed by an increase in aeroallergens and air pollution (57%), UV radiation exposure (29%), mold in houses (24%) food security (14%) and mental health issues (10%) [70]. 

A recent comprehensive analysis of adaptation mechanisms addressing infectious diseases in national adaptation plans in OECD countries found significant differences between countries which face similar risks in number and quality of adaptation measures [71]. However, there were also similarities between countries, specifically in the negligible reference to vulnerable groups and local risks, and to implementation aspects including budget and evaluation. Furthermore, it was found that infectious diseases are typically addressed by sector at the expense of a more holistic, multi-disciplinary, cross-sectoral approach, and by mainstreaming adaptation measures into existing public health projects rather than developing separate initiatives. 

The gaps in adaptation strategies between countries are also evident in a 2015 report of the WHO on “Implementing the European Regional Framework for Action to protect health from climate change” [72]. Within the Mediterranean region, out of eight topics (governance, vulnerability, adaptation strategies, mitigation, strengthening health systems, raising awareness and building capacity, green health services and sharing best practice), Turkey reached full score in 1 topic, Italy in 2, and Spain in 7. While this report covered the impact of climate change on health generally, and not specifically VBD, it is evident that the quality of adaptation policy vary greatly between countries in the WHO-Europe region. 

We reviewed the adaptation documents of Mediterranean countries, and selected countries to represent the geographical, climatic and socio-economic diversity of the basin (Spain, Italy, Malta, Turkey, Israel, Egypt) as presented in Table 1. It is important to note that our selection was limited to countries that have substantial adaptation plans or policies, while many countries still lack them [73]. We searched the websites of the Ministry of Health and Ministry of Environment in each country, as well as google search engine, using the keywords “climate change”, “adaptation”, “health” and “vector-borne diseases”, and the name of the country. We looked for national adaptation plans and national reports, in five languages known by the researchers (English, Spanish, Italian, Hebrew and Arabic). To reflect the geographical diversity of the Mediterranean, we included also Turkey and Malta (although these languages are not their formal languages) where we found comprehensive English materials. To the best of our knowledge, this study contributes to comparisons of adaptation plans [71,74] in the literature in countries which are less assessed in the literature due to language limitations. 

The UNEP Handbook on Methods for Climate Change Impact Assessment and Adaptation Strategies [75] proposes a typology of adaptation measures to protect human health from climate change. These include five types of measures: (1) surveillance and monitoring, (2) infrastructure development, (3) public education, (4) technological or engineering strategies and (5) medical interventions.

Table 1 presents the types of adaptation measures in selected Mediterranean countries according to the UNEP criteria [75], with modifications to the case of VBDs: infrastructure development (excluding health systems infrastructure) and technological and engineering strategies were modified to environmental management, and medical interventions, including health-related infrastructure, modified to health system preparedness. This category now includes all health system preparations: infrastructure, capacity building, and interventions.

Panic and Ford [71] developed a detailed typology of adaptation for infectious-diseases, to analyze national adaptation plans in OECD countries, which includes 17 different types of adaptation measures. However, we chose the much more general UNEP criteria, since most of the Mediterranean countries we surveyed did not have the detailed resolution of VBD-related adaptation measures that many OECD countries now have for infectious diseases in general. 

As evident from Table 1, all six countries reviewed have adaptation policy documents: Spain, Malta, Turkey, and Egypt have adaptation plans or strategies, Italy has a climate change report and Israel has policy recommendations, which have not yet been adopted by the government. All countries designed policy mechanisms to reduce the emerging risks of VBDs due to climate change, and almost all of them address the four types of policy mechanisms analyzed: monitoring and surveillance, environmental management, health system preparation and public education.

However, the variance between different countries is large. All countries we reviewed plan to enhance monitoring and surveillance, but with different emphases. Spain, Malta, Israel and Egypt plan integration of environmental data (vectors) and health (morbidity and mortality) data, which is important in order to assess the patterns of disease outbreaks. Spain, Malta, Turkey and Israel include modelling of future scenarios in their adaptation plans, and similarly Turkey, Spain and Italy include identification of areas at risk or increasing monitoring in such areas. 

Other mechanisms are less prevalent, for example, Turkey emphasizes a focus on the vulnerable group of seasonal agriculture workers, while other countries did not identify particular populations at risk for VBDs in their plans. Israel includes the aspect of monitoring at border areas, which is especially important to this small state, but relevant also to other countries, and several countries acknowledged the importance of international collaboration, important specifically regarding vectors, who know no borders. Italy intends to develop a plan for addressing cross-border threats, Turkey recently harmonized with international disease networks, and Egypt plans to improve its regional and international communication to identify pathogens and infections, and develop prevention strategies. 

Regarding environmental management, while all countries plan a prevention system, Spain and Italy further detail inspection and quarantine of products coming from endemic zones, and Israel aims to improve vector management regulations for local authorities. As for the health system, almost all countries identify the need to develop early warning systems and response plans. 

Another overarching measure is training health professionals, although emphases are different across countries: Spain suggests that health workers and epidemiologists should get a global perspective on VBDs, and that practitioners need multi-disciplinary training, including entomology and tropical medicine. Italy includes identifying priority prevention. Turkey expands training to the professions of emergency, provincial, protective and family health workers, and Israel refers to medicine and nursing students and generally public service workers, especially in areas at risk. 

The multiplicity of professional backgrounds, both within the health system and across other sectors, means that the relevant staff have multiple professional languages (epidemiology, public health, entomology, climatology *etc.*), which need to be bridged in a multidisciplinary manner to enhance collaboration [76]. 

Other measures of preparedness of the health systems are inconsistent across countries. While the Turkish need to form regional diagnosis laboratories may not be relevant to other countries that already have such infrastructure, the Israeli suggestion to conduct annual preparedness exercises and the Maltese and Spanish identification of the need for risk assessments are relevant to other country contexts [77]. Finally, regarding public education, most countries mention this aspect of adaptation very briefly, noting campaigns and raising awareness. Public participation, which can improve implementation of policies and programs [78,79], is largely absent from the adaptation policies. 

Bearing in mind that many countries in the region still do not have such plans, the key points that stem from comparing the VBD policy measures in six Mediterranean adaptation plans can be summarized as follows: VBD-related adaptation measures are present in all plans, including the major aspects of surveillance and monitoring, environmental management, preparation of health systems and public education. Nevertheless, the practicalities of implementing these measures differ greatly between countries, with some countries detailing surveillance measures such as identifying areas and populations at risk, while others only mention generally “strengthening surveillance”. This resonates with the recent analysis of adaptation plans in OECD countries [71], which found that most do not identify populations at risk. 

Similarly, all countries plan to improve vector management, but only a few detail regulatory aspects such as inspection and quarantine, or designating responsibilities to local authorities. The situation is better regarding health system preparedness, with countries stating specific measures such as early warning systems and training, but here too, the differences between countries cannot be tracked only to their geographical and societal differences, and countries can learn from the adaptation plans of their counterparts. 

The weakest aspect of the adaptation plans in general is probably that of public education, which is very limited. WHO recognizes the need to improve not only information to the public, but actually engage the public at each step of policy-making and implementation, and this aspect is missing from all Mediterranean adaptation policies we reviewed. 

Finally, most countries did not detail the agencies responsible for implementation and the collaborations between them. Israel, for example, did designate specific responsibilities to the Ministry of Health, Ministry of Environmental Protection, Nature and Parks Authority, Ministry of internal Affairs and local authorities but did not specify how the agencies will coordinate. Turkey did note a collaboration to fight vectors between the Ministries of Health, Environment and Urban Planning, and Forestry and Water Affairs, and Italy emphasized coordination and integration across government and disciplines to implement prevention interventions, including multi-disciplinary technical workshops. The limited and partial reference to inter-sectorality corresponds with the recent review of adaptation plans in OECD countries, which were mostly sectoral rather than holistic.

**Table 1:** VBD adaptation mechanisms in national adaptation plans and policies in Mediterranean countries.

Country	Adaptation Documents (and Drafting Agency)	Surveillance and Monitoring	Environmental Management	Health System Preparation	Public Education	Cross-Border Aspects
Spain	Impacts on Health of Climate Change—Executive summary 2013 [80] (Ministry of Health, Social Services and Equality) Global Change Spain 2020/2050, 2012 [81] (Complutense Center for Environmental Studies and Information) PNACC Spanish National Climate Change Adaptation Plan, 2008 [82] (Minister of Environment and Rural and Marine Affairs)	Climate modeling & future scenarios Surveillance of VBD and climate data Proactive epidemiological surveillance in areas where transmission is detected Consolidate ecological monitoring networks	Inspection, certification and quarantine of commercial products coming from endemic zones Establish a prevention system with comprehensive vector-control programs	Early warning systems in public health sector Medical practitioners and epidemiologists to acquire global perspective on VBD and factors involved Practitioners’ training should include support from medical entomology and tropical medicine.	Establish preventive campaigns based on climate and diseases data Inform public and tourists on preventive measures they can take, and daily actions to control vectors	
Italy	National Prevention Plan 2014-2018 [83] (Ministry of Health) Italy Climate Change Report [84] (Ministry for the Environment, Land and Sea) 5th national communication to the UNFCCC, 2009 [85] (Ministry for the Environment, Land and Sea)	Constant monitoring of population at risk Initiatives to survey and monitor increase of disease vectors Regional programs of active surveillance for co-infection of HIV/leishmania	Environmental interventions coordinated with early warning systems Quarantine of imported goods	Develop risk-based, early warning systems regarding vectors and pathogens Develop a preparedness and response plan Improve identification abilities and quick emergency response Train health professionals in identifying priority prevention	Communication of adaptation policy to public Information campaigns	General plan for serious cross-border health threats of biological origin
Malta	National Climate Change Adaptation Strategy, 2012 [86] (Ministry for Resources and Rural Affairs) National Seminar on Health Effects of Climate Change—Raising Awareness and Building Capacity 2009 [87] (Department of Health Promotion and Disease Prevention)	Strengthen surveillance of diseases and vectors Link environmental and epidemiological data Further assessment of climate change impacts on individual vectors	Improve early identification and response	Undertake a risk assessment Identify measures to reduce possibility of outbreaks Develop a plan for control during outbreaks Address gaps in entomological expertise Enhance diagnostics	Education campaigns to focus on health impact of climate change, and aim at changing behavior	
Turkey	National Climate Change Strategy, 2010–2020 [88] (Ministry of Environment and Forestry) National climate change adaptation strategy and action plan 2011 [89] (Ministry of Environment and Urbanization)	Strengthen surveillance Contagious Diseases Monitoring and Control System Project—A regional project focusing on a vulnerable population of seasonal agricultural workers Identify areas at risk, screen populations and plan constant monitoring Forecast changes in disease patterns	Strengthen vector control	Form regional tropical diseases diagnosis laboratories and strengthen public health laboratories Develop emergency response action plans and pilot programs in areas at risk. Train health professionals, including emergency, provincial, protective health and family health workers Research on effectivenessof adaptation measures taken in health sector Increase capacities of health organizations in risky areas	Guides and training to citizens Publishing the adaptation plan nationally	Recently harmonized with international disease networks, and aligned regulation on surveillance and control of communicable diseases to EU legislation Strengthen cooperation with countries and international organizations on climate change and health generally
Israel	Israel Climate Change Information Center, report No. 2: Policy recommendations, 2012 [90] (Ministry of Environmental Protection, not adopted by government)	Develop regular monitoring of vectors (appearance, density, geographical spread) and analysis of samples of vectors for pathogens Specific monitoring at border areas Surveillance of VBD-related morbidity and mortality Forecast which vectors and hosts may pose risk due to climate change Monitor vector control	Strengthen local authorities regulations for vector control Treat and prevent “standing” water	Health system to prepare for outbreaks: identifying early symptoms, treating sick population, annual preparedness exercise Develop guidelines for health professionals, including medicine and nursing students, and generally for public service workers, especially in areas near vectors’ breeding sites	Public alerts in case of outbreaks Public notification regarding prevention Guide public to spray vectors in private properties and report vectors and vector habitats in public properties	
Egypt	National Strategy for Adaptation to Climate Change And Disaster Risk Reduction 2011 [91] (The Egyptian Cabinet: Information & Decision Support Center) 2nd national communication to UNFCCC, 2010 [92] (Ministry of State for Environmental Affairs; Egyptian Environmental Affairs Agency)	Develop a surveillance system for infectious diseases and disease vectors Develop early detection Establish an accessible integrated database with information exchange between all concerned parties	Ongoing control of mosquitoes	Develop early warning systems and control programs for infectious diseases Improve access to quality health services Develop research: epidemiological, vectors migration, prevention, biological vector control, monitoring. Malaria may be a threat due to climate change, and international experience in malaria control should be implemented, including early diagnosis, prompt treatment, preventive measures, building research capacity	Raise awareness on pro-active health measures and on vectors and parasites	Develop regional and international communication to identify pathogens and infections and develop prevention

## 4. The Need for Cross-Border Collaborations regarding VBDs 

The expansion patterns of VBDs know no political borders, and efficient prevention of climate-induced spread of VBDs must include both national actions and international cooperation. In recent years, international cooperation proved successful in reducing the burden of VBDs across the world, such as Chagas disease in South America [93] and Malaria in Africa [94]. In the Middle East, Jordan, Israel and the Palestinian Authority collaborate to combat Leishmaniasis [95]. 

The emergence and spread of VBDs in recent years led to increased international monitoring cooperation. For example, in 2014 the European Food Safety Authority (EFSA) and the European Centre for Disease Prevention and Control (ECDC) established VectorNet: a European network for sharing data on the geographic distribution of arthropod vectors, transmitting human and animal disease agents. Similarly, the US Center for Disease Control and Prevention (CDC) operates ArboNet, a national surveillance system combining data from all US states. The WHO Western Pacific conducted a regional project on strengthening control of VBD to lessen the impact of climate change [96], with six outputs:

1. Increased awareness and involvement of communities and stakeholders within and beyond the health sector in actions to minimize VBD consequences due to climate change
2. Strengthened surveillance for vector-borne infections and climate change and capacity for rapid response to VBD outbreaks
3. Strengthened capacity for vector control
4. Strengthened capacity for effective diagnosis and treatment of VBDs
5. Strategic information on knowledge gaps generated and utilized to better respond to climate change-induced VBDs
6. Strengthened country programs and effective and efficient project management

Climate change mitigation and adaptation also require cross-border cooperation, and regional networks have been established globally. In the Mediterranean region, the European Climate Adaptation Platform (Climate-ADAPT) supports European countries including European Mediterranean countries, and the ClimaSouth project supports the non-European countries of the southern Mediterranean. Both of these projects are EU funded. 

The need for regional cooperation is especially important in such an ecologically sensitive and highly populated region as the Mediterranean. The region is also characterized by high socio-economic diversity between countries and by political tensions, particularly between Israel and Arab countries but also between other countries such as Greece and Turkey. These political tensions make regional cooperation very challenging. 

In what follows we examine two models for a productive Mediterranean cooperation, the Mediterranean Action Plan (MAP) and EpiSouth, with the aim of recommending future collaboration based on the strengths of each model, in the following section.

The MAP is a multilateral plan adopted in 1975 by the Mediterranean countries to protect the Mediterranean Sea, of which the Barcelona Convention for Protection against Pollution in the Mediterranean Sea serves as the legal platform. In the setting of the high socio-economical diversity and continuous conflicts of the Mediterranean parties, this mechanism enables regional cooperation of all Mediterranean countries in Europe, the Middle East and Africa [97]. 

In the framework of MAP, all member countries established marine pollution monitoring and reporting systems for industrial pollution, and monitoring capacities improved. However, establishing a comprehensive long-term regional monitoring system still requires more investment, especially in the countries on the southern and eastern shores [98]. The greatest strength of MAP is also its main challenge: creating cooperation between highly diverse countries. The fact that all members are connected directly to the MAP Secretariat enables productive cooperation despite gaps and conflicts. For example, under the umbrella of MAP, Israel strengthened an environmental cooperation with Egypt and signed a sub-regional agreement with Egypt and Cyprus in 1995 [99]. Yet, there is significant variation in the capacity of diverse countries to implement the MAP protocols. 

MAP provides financial and capacity-building incentives to less developed countries, but there are no sanctions against parties that do not meet their commitments. This sanction-free policy encourages countries to participate, but means implementation cannot be enforced. While in many cases MAP has contributed to national legislation and enforcement, and in turn to a cleaner environment, its overall success in actual improvement of the marine environment and ecosystems is questionable [98,99,100]. Even in problematic areas that improved dramatically, such as waste water treatment and sanitation infrastructure, there are major gaps between the situations in the northern and southern shores and it is not always clear whether improvement was achieved thanks to the MAP or due to other factors [101,102]. Moreover, there are still major gaps in both monitoring and reporting [98] and one of the criticisms of MAP is that it lacks clear targets and evaluation measurements [102].

In contrast to the MAP, the EpiSouth project has a less stable framework, which depends on European Union funding in cycles of four years. The first phase, “A Network for Communicable Disease Control in Southern Europe and Mediterranean Countries”, was active in 2006–2010. The second phase, EpiSouth Plus, established “The Network for the Control of Public Health Threats and other bio-security risks in the Mediterranean Region and Balkans” in 2010–2013. 

The final evaluation report of the second phase, EpiSouth Plus, concluded that the project was valuable in creating a network of experts from all Mediterranean countries, and enhanced communication and collaboration particularly between European, Asian and African countries. The project improved surveillance, health professionals' capacities, preparedness of health systems and cross-border data sharing. However, it had significant weaknesses, most importantly for the Mediterranean context, by not considering the needs of non-European countries, which were consequently less active in the project [103]. 

Now in its third phase, the project is entitled MediLabSecure and aims at “preventing vector borne diseases around the Mediterranean and Black Sea Regions by creating new networks” in 2014–2017. The current phase is aimed solely at non-European countries in the Mediterranean and Black Sea, and particularly at integrating surveillance and monitoring, training public health experts and improving preparedness, and conducting risk assessments [104]. 

## 5. Recommendations 

The diverse countries we surveyed for VBD adaptation include in their policies all the relevant basic adaptation mechanisms: monitoring and surveillance, environmental management, health system preparedness and public education. There is variance across countries, which can only partially be attributed to variance in VBD risks, and in many cases a country detailed measures relevant to its counterparts. 

The experience of the regional collaboration projects we reviewed shows the limited capacity of Asian and African Mediterranean countries to plan and implement environmental and health policies, which comes as no surprise considering the extensive socio-economic gaps in the basin. Nevertheless, the European countries we examined also proposed a relatively basic and sectoral approach, which is consistent with the literature on ad hoc and fragmented adaptation policy elsewhere [71]. 

Considering that albeit tremendous investment there are no vaccinations available for the VBD that emerge in the Mediterranean due to climate change, countries should be well prepared and collaborate to fight this threat to public health. Based on our findings and on other regional and international adaptation documents [75,96], we propose recommendations in the following order: basic national recommendations, advanced national recommendations and regional recommendations for the Mediterranean basin.

On the national level, our basic recommendations are that countries should improve the policy mechanisms already included in their adaptation plans. Specifically:

1. *Monitoring and surveillance* should include regular monitoring of emergence, density and geographical distribution of vectors that could be hosts and pathogens, and a representative sample of the population of these vectors should be checked continually for the presence of all their possible pathogens. Epidemiological data on VBD-related morbidity and mortality should be collected systematically, and consolidated with environmental and ecological data.
2. *Environmental management* should be detailed, and the Spanish and Italian examples of inspection and quarantine, or the Israeli example of designating responsibilities to the local level are a start in this direction that is relevant to other countries. Considering that countries have legal ways to improve vector control, such as requiring individuals to eliminate breeding sites in their living areas [77], the current general statements on enhancing vector management could be improved.
3. *Health system preparedness* for dealing with outbreaks of VBDs should be evaluated annually, specifically before and during the mosquito breeding and activity season. The diagnostic capability for the different pathogens that might reach the Mediterranean basin should be checked and exercised annually by trained personnel in equipped laboratories, particularly before the breeding and activity season of the potential vectors and hosts. Also, health system should deal with the issue of screening blood donations for pathogens. Regarding training, each country identified different target groups in the health sector and different content; countries can learn from their counterparts to improve this aspect. Moreover, countries should conduct risk assessments and identify areas at risk, and populations at special risk for VBDs, as Turkey did in the example of agriculture workers. Countries should also include immigrant populations such as refugees and migrant laborers in their assessments, as this is a particularly vulnerable group in the Mediterranean, and clear policies should exist regarding their screening and treatment.
4. *Public education* should be strengthened in all countries, and aim to involve the public in combating and preventing VBDs through means of identifying, reporting and managing breeding sites, and information about individual protection in daily routines and in case of an outbreak. The public can significantly reduce its exposure risk by taking simple measures such as eliminating small breeding sites and using mosquito traps and mosquito nets; countries should define target groups (as Spain did with tourists) and design appropriate education campaigns and curriculums.
5. *Evaluation and assessment* of implementation of adaptation plans should be conducted, in order to identify best practice, which is very limited today in adaptation policy generally, and regarding VBDs specifically [71].

As to the more advanced recommendations for the national level, countries can benefit from adopting the ecosystem approach. According to the Millennium Assessment [105,106] the relevance of the ecosystem approach to VBD management is threefold:

1. *Integrated vector management*, including biological pesticides and technologies. This prevents not only VBDs, but also negative effects of chemical pesticides on public health (due to spraying and mosquito repellents) and on the local ecosystems (due to spraying and management of water resources such as wetlands).
2. *Public participation*, which is relevant to VBD management in the culturally diverse Mediterranean region. Public participation can contribute local knowledge on breeding sites and environmental friendly solutions; involving the public can also promote community-based vector control and community based-surveillance [96]. Moreover, the public should be involved in prioritizing adaptation and in determining methods for environmental management and preparedness of the health system, which is funded by the public and aims to serve the public.
3. *Trans-disciplinary work*. Vector management requires inter-sectoral cooperation between health, climate, environment and development government agencies, and between government and the private sector, civil society and academia. Vector management also needs to include all levels of government: local, national and international. Specifically, the responsibilities should be clarified between local authorities and central government. Such trans-disciplinary work was not evident in the adaptation policies we reviewed, which were typically written by one agency, and designated responsibilities mainly to the environment and health agencies. Countries should work to enhance collaboration across these agencies and development and production agencies such as economy, agriculture, energy and planning, whose work influences mosquito breeding sites and vector hosts. In addition, it should be clear which agency is responsible for overseeing implementation of adaptation, in order to avoid delays and duplications.

On the Mediterranean level, the climatic and political characteristics of the region require cooperation to prepare for possible emergence of VBDs due to climate change. However, the conflicts and wide socio-economic gaps in the area challenge a direct cooperation between countries. 

Despite the regional difficulties, MAP is an example of continuous regional environmental cooperation for almost 40 years. Similarly, EpiSouth is a more recent testament that regional cooperation is also possible in the health sector. 

Therefore, we suggest that health-related adaptation to climate change in the basin should be under the umbrella of an international, permanent and neutral body. This is particularly crucial in the case of VBD since mosquitoes and ticks may spread easily across political borders toward populated areas. Moreover, we suggest that rather than creating duplications, existing frameworks can be utilized, such as MAP or EpiSouth, which both have the capacity and experience to enhance the basic adaptation mechanisms required. 

Most importantly in the context of the Mediterranean basin, and due to the limited success of both initiatives in Asian and African Mediterranean countries, one of the main objectives should be to design and sustain a framework that is equally accessible and relevant to all parties, in terms of existing infrastructure, capacities, culture and language. VBD outbreaks in one country are a threat to all its neighbors, and it is in the interest of all countries to participate in mutual learning and assist one another.

## 6. Conclusions

The Mediterranean region is vulnerable to climatic changes. During the last decades, a significant warming trend was measured in the basin; extreme weather events have become more common with an increase in the frequency and severity of heat waves, parallel with a reduction in rainfall amounts. As a result, it is expected that VBDs will be influenced by climate change. For some diseases (such as West Nile virus) this was proved recently [30] and for others (such as dengue and Chikungunya) the risk for local transmission related to climate change in the near future is real. Therefore, climate change adaptation and preparation for changing patterns of VBD distribution in the Mediterranean basin is essential. 

Our analysis of six representative Mediterranean countries shows that they have started preparing for this threat, but preparation levels vary and remain basic in all country cases. Moreover, regional-level policy is limited and temporary, and depends on international organizations. We make basic and advanced recommendations for countries of this region to improve their adaptation plans and further recommend enhancing regional collaboration in order to address cross-border aspects of vector transmission. Based on the achievements and drawbacks of existing regional frameworks in advancing national legislation and regional cooperation despite the challenges in the region, we suggest that a neutral and stable framework should also address the environmental health aspects of climate change, particularly the risk of VBD transmission since the vectors of infectious diseases know no political borders. 

Since past experience shows significant gaps in implementation of regional frameworks between European, Asian and African countries, this network must be geared towards Asian and African capacities and characteristics, thereby simultaneously protecting the public health of European countries. Finally, climate change is already here, therefore the Mediterranean countries should advance regional vector management systems before VBDs develop into regional outbreaks.
